# Psychological health of family caregivers of children admitted at birth to a NICU and healthy children: a population-based cross-sectional survey

**DOI:** 10.1186/1471-2431-4-24

**Published:** 2004-12-14

**Authors:** Anne F Klassen, Shoo K Lee, Parminder Raina, Sarka Lisonkova

**Affiliations:** 1 Centre for Community Child Health Research, Department of Pediatrics, University of British Columbia, Vancouver, BC, Canada; 2 Centre for Healthcare Innovation and Improvement, Dept of Pediatrics, University of British Columbia, Vancouver, BC, Canada; 3 Evidence-Based Practice Centre, Dept of Clinical Epidemiology and Biostatistics, McMaster University, Hamilton, ON, Canada

## Abstract

**Background:**

There is little information in the research literature on how parents of children who spend time in a neonatal intensive care unit (NICU) adapt psychologically to the demands of caregiving beyond the initial hospitalization period. Our aim was to compare parents of NICU children with parents of healthy full-term children, looking specifically at the relationship between parental psychosocial health and child characteristics, as well as the relationship between important predictor variables and psychosocial health.

**Methods:**

A cross-sectional survey was sent to parents as their child turned 3 1/2 years of age. The setting was the province of British Columbia, Canada. The sample included all babies admitted to tertiary level neonatal intensive care units (NICU) at birth over a 16-month period, and a consecutive sample of healthy babies. The main outcome was the SF-36 mental component summary (MCS) score. Predictor variables included caregiver gender; caregiver age; marital status; parental education; annual household income; child health status; child behavior; birth-related risk factors; caregiver strain; and family function.

**Results:**

Psychosocial health of NICU parents did not differ from parents of healthy children. Child health status and behavior for NICU and healthy children were strongly related to MCS score in bivariate analysis. In the pooled multivariate model, parental age, low family function, high caregiver strain, and child's internalizing and externalizing behavioral symptoms were independently associated with lower psychosocial health. In addition, female gender was associated with lower psychosocial health in the NICU group, whereas lower education and child's problem with quality of life indicated lower psychosocial health in the healthy baby group.

**Conclusions:**

Overall, parental gender, family functioning and caregiver strain played influential roles in parental psychosocial health.

## Background

Neonatal intensive care is associated with a range of long-term health problems such as cerebral palsy, mental retardation, deafness, blindness and milder but more common problems such as learning disabilities and behavioral problems [1-13]. Although these problems create challenges for the parent responsible for the day-to-day provision of care to their child at home, the impact of caregiving on the health of parents of children discharged from neonatal intensive care units (NICUs) remains an under-explored research topic. There is a literature that focuses on the early hospitalization period. These studies show that mothers of preterm infants experience more severe levels of psychological distress in the neonatal period than do mothers of healthy full-term infants [14-17]. In the few studies that compare the impact of caregiving on parents of children discharged from NICUs with parents of healthy full-term children, the addition of a preterm infant into the family has been shown to have negative repercussions for the family in some studies [18-21], but not in others [22-24]. In one of the few NICU studies where parental mental health was the primary outcome measure, mothers of high and low-risk very low birth weight infants were compared with parents of healthy full-term infants [17]. The authors report that early differences between the groups at one month and two years were no longer apparent by the age of three, although parenting stress remained high throughout.

In the present study, we sent a questionnaire booklet to mothers of all children admitted to a level III neonatal intensive care unit in the province of British Columbia (Canada) over a 16 month period to collect data on a range of factors in order to examine both neonatal and caregiver outcomes. Our study differs from other NICU follow-up studies in that it is population-based, focuses on preschool aged children and examines the full spectrum of NICU graduates. The aims of this paper are two-fold: (1) to compare psychosocial health of parents of NICU children with parents of healthy full-term children, looking specifically at the relationship between parental psychosocial health and child characteristics (i.e., health status, behavior problems, and birth-related risk factors); and (2) to identify predictors of parental psychosocial health (i.e., socioeconomic and demographic variables, child characteristics, caregiver strain, and family function).

## Methods

### Sample

Ethical approval was gained from the University of British Columbia and participating hospitals. Our NICU sample included 2221 surviving babies admitted for more than 24 hours to one of three level III NICUs in British Columbia (BC), Canada over a 16 month period (March 1996 to June 1997). These 3 hospitals (Children's and Women's Health Centre of BC, Royal Columbian Hospital, Victoria General Hospital) provided 100% of the tertiary care NICU beds in the province. The birth mothers' name and contact details were obtained from the health records department at two hospitals and manually extracted from ledgers of the third hospital. Our list of babies was matched with provincial mortality records to exclude any babies that had died after discharge from the NICU and thereby prevent questionnaires being sent to bereaved parents.

A comparison group of 718 healthy singleton full-term babies was recruited from the two hospitals with a hospital-based primary care unit (i.e., Children's and Women's Health Centre of BC and the Royal Columbian Hospital). This sample included all babies delivered over an 11 month period (March 1996 and January 1997) by primary care physicians at these two clinics. Babies with a sibling in the NICU sample and babies subsequently admitted to an NICU for more than 24 hours were excluded. Contact details for the mother were obtained from the health records department at one hospital, and directly from the primary care unit at the other.

We excluded from the sample 150 babies (123 NICU; 27 healthy children) who did not meet our inclusion criteria for the following reasons: parent did not speak English (n = 95); baby died (n = 34); mother died (n = 6); and not applicable (n = 1). In addition, we excluded cases where the questionnaire was completed on the wrong child (n = 7) and where a comparison baby was subsequently admitted to a NICU (n = 7). The overall response rate (after exclusions), was 55% (54.3% NICU, 56.9% healthy baby group). The response rate for located families (82.8% of the sample was located) was 67.4% (n = 1140) for the NICU group, and 66.4% (n = 393) for the comparison group. Five NICU respondents returned a signed consent form without a completed questionnaire and were dropped from the analysis. Seventy-five percent of parents provided permission for data linkage between the questionnaire data and CNN database. The NICU sample included 181 children that were part of a multiple birth group: 171 twins; and 10 triplets. Table 1 contains sample characteristics. Most questionnaires (98%) were completed by a biological parent, most often the mother (96%). The NICU sample was composed of 1.8% fewer biological parents; 2.6% more male respondents, and 11.9% more families who earned less than $50,000 per year.

**Table 1:** Characteristics of study sample

	Group; no. (%) of subjects	
	NICU N = 1135	Comparison N = 393

Biological parent^1^	1091 (97.7)	389 (99.5)
Female^1^	1070 (95.4)	383 (98.0)
Married/common-law	962 (85.9)	344 (87.8)
*Age of parent, years*		
19–29	195 (17.8)	61 (15.7)
30–39	704 (64.1)	265 (68.3)
≥ 40	199 (18.1)	62 (16.0)
*Education level*		
University	373 (33.4)	146 (37.4)
Trade/technical school or community college	494 (44.3)	176 (45.1)
High school graduation	185 (16.6)	50 (12.8)
No high school diploma	64 (5.7)	18 (4.6)
Household income, $^2^	511 (48.1)	136 (3)
<30,000	247 (23.3)	58 (15.5)
30 – 49,999	264 (24.9)	78 (20.8)
50 – 79,999	333 (31.4)	145 (38.7)
80 >	218 (20.5)	94 (25.1)
Male children in the sample	633 (55.8)	198 (50.6)
*Age of child, years*		
3 years	784 (69.3)	253 (64.4)
4 years	328 (29.0)	134 (34.1)
5 years	19 (1.7)	6 (1.5)

^1^p < .05 (chi-square, Fischer's exact test); ^2^p = .0018 (chi-square)

### Materials

Our main measure of outcome was the SF-36 mental component summary (MCS) score [25,26]. The SF-36 is a well validated generic measure of adult physical and psychosocial health related quality of life (HRQL), which is composed of 36 items that measure 8 health domains. The MCS is computed from the following four domains: mental health (5 items); vitality (4 items); social functioning (2 items); and role limitations due to emotional problems (3 items). It has a mean of 50 and standard deviation of 10 and represents the mean and standard deviation of the general population (USA).

Child health status was measured using the Health Status Classification Preschool Version (HSCS-PS) [27]. This measure asks about twelve health status (HS) problems that we have grouped into the following 4 categories: neurosensory (i.e., seeing and hearing); motor development (i.e., getting around, using hands and fingers, taking care of self); learning (i.e., speaking, learning/remembering and thinking/solving problems); and quality of life (i.e., pain/discomfort, feelings, behavior and general health). Each attribute has 3 to 5 levels of severity ranging from normal function to severe functional limitations. For each category of health problems, we recoded the data into the following: no problem; a mild problem; or a moderate or severe problem.

Child behavior was measured with the Child Behavior Checklist 1.5–5 (CBCL/1.5–5) [28]. This questionnaire measures internalizing, externalizing and total problems, and scales can be scored categorically to indicate normal, borderline or clinical range scores.

Data for birth-related risk data were obtained from the Canadian Neonatal Network Study [29] for the NICU children whose parents provided written consent for data linkage. The following variables were examined: birthweight; gestational age; small for gestational age; multiple birth, apgar score less than 7 at 5 minutes; congenital anomalies; the presence of a major morbidity (i.e., a composite score for the presence of at least one of the following: chronic lung disease (at 36 weeks); severe intraventricular hemorrhage (≥ grade 3); nosocomial infection; necrotizing enterocolitis; retinopathy of prematurity (≥stage 3)); and neonatal illness severity score [30].

Caregiver strain was measured using the Parental Impact-Time (PTT) scale from the Infant Toddler Quality of Life Questionnaire [31]. This 7-item scale asks parents to indicate limitations in the amount of time in the past 4 weeks they had for their own personal needs due to problems with their child's health (e.g., physical, emotional, cognitive, behavior, temperament). Scores on these scales can range from 0 to 100, with lower scores indicating greater caregiver strain.

Family function was measured using the Family Assessment Device (FAD) [32]. Scores for this 12-item questionnaire can range from 0 to 36, with higher scores indicative of greater family dysfunction.

### Procedure

A questionnaire booklet, which included the questionnaires described above, was sent to the address of the birth mother as her child turned 3 1/2 years of age. A consent letter was included to obtain permission to link the questionnaire data with hospital birth records. The primary caregiver in our study was defined as the person who, to that point in the child's life, had spent the most amount of time with the child. This could include the mother or father or another parent (e.g., grandparent, foster parent, guardian). We asked the primary caregiver (referred to in this paper as parent) to complete the questionnaire booklet and consent form. Non-respondents were sent a reminder letter, additional copies of the questionnaire booklet and a phone call as necessary. If the telephone number was not in service or reassigned, or a questionnaire booklet was returned to us from the post office, we implemented a comprehensive search strategy that involved searching the Internet and contacting the mothers' primary care physician.

### Data analysis

To address the first objective, we compared the psychosocial summary score for the SF-36 questionnaire for parents of NICU children and parents of healthy children using student's *T*-test. *T*-test, ANOVA and the equivalent nonparametric tests, and Spearman correlation were used to explore relationships between MCS score and various child characteristics, including health status, behavior and birth-related risk factors. For health status and child behavior, we computed an effect size (mean difference divided by standard deviation of the group with no problems (health status) or with scores in the normal range (behavior)), to look at the magnitude of the difference in MCS score between subgroups for the NICU and healthy baby samples, and used the Cohen's guidelines for interpretation (0.2 is small, 0.5 is medium, 0.8 is large) [33].

To address the second objective, multiple regression analysis was used to examine the independent effects of, and proportion of variance in MCS scores explained by our predictor variables. For the analysis we examined a pooled model and a model where we stratified by group membership (i.e., NICU vs. healthy baby sample) to separately examine the contribution of each predictor variable for the two samples. Variables with significant (p < .05) or borderline p-values in bivariate analysis were included in the model. Certain birth-related risk factors (i.e., birthweight, congenital anomalies, illness severity score, and gestational age) were entered into the model on the basis of clinical rather than statistical importance, however, no effects were found. Potential predictor variables include the following: caregiver's gender; caregiver's age (continuous); marital status (married or common-law versus other), caregiver's education (less than high school graduation vs. other); annual household income (< or > $30,000); child health status (i.e., neurosensory; motor development; learning; and quality of life problems); child behavior; caregiver strain (continuous); and family function (continuous). For child health status and behavior variables, no problem (health status) and scores in the normal range (behavior) were the reference categories, with mild and moderate/severe (health status) or borderline and clinical range scores (behavior) entered separately, or combined and entered as dichotomous variables. We computed effect sizes to interpret the significance of beta coefficients.

## Results

### Psychosocial health comparing NICU and healthy children

The unadjusted mean MCS score for parents of NICU children did not differ from parents of healthy children (48.2 versus 48.8; p = .305). We also compared MCS scores after adjusting for the three sample characteristics that differed between the two groups (i.e., proportion of biological parents; gender of subject; and those with lower household income), and no differences were found in the outcome variable.

### Psychosocial health by child health status problem

On the HSCS-PS, 55.2% of healthy children had no health problems in any area, compared with 39.8% of NICU children (p < .001 on Chi-square). Table 2 shows the joint distribution of health status problems across the four categories for the NICU and healthy sample. These results show that the NICU sample had a higher proportion of children with more health status problems, as well as a higher proportion with moderate/severe versus mild problems.

**Table 2:** Distribution of children with health status problems across the 4 health status categories for NICU and healthy children

HSCS problems by domains		NICU (N = 1104)	Comparison (N = 386)
no problem	N	438	215
	%	*39.67*	*55.7*
1 mild problem	N	309	111
	%	*27.99*	*28.76*
2+ mild problems	N	183	37
	%	*16.58*	*9.59*
1 moderate/severe problem only	N	40	7
	%	*3.62*	*1.81*
1 moderate/severe problem + any mild	N	69	15
	%	*6.25*	*3.89*
2–3 moderate/severe problems	N	60	0
	%	*5.43*	*0*
4 moderate/severe problems in all domains	N	5	1
	%	*0.45*	*0.26*

p < 0.0001, chi-square

For parents of NICU children, for all 4 health status categories, parental MCS scores decreased as severity of the child health problem increased (see Table 3). Effect sizes comparing parents of children with no health status problems with parents of children with a moderate or severe health status problem were all moderate to large indicating important differences in parental mental health according to Cohen's benchmarks. The results for parents of healthy children show similar trends, with mainly moderate to large effect sizes.

**Table 3:** Parental mental health summary score, 95% confidence intervals, number of subjects, p-value and effect size for child health status category

Sample	Type of HS problem	None	Mild	Moderate or Severe	p-value*	Effect size
NICU children	Neurosensory	48.4 (47.7, 49.0) n = 975	46.3 (41.7, 51.0) n = 33	41.9 (36.0, 47.8) n = 17	.023 *.040*	.63
	Motor development	49.1 (48.5, 49.8) n = 789	45.8 (44.0, 47.6) n = 174	42.0 (38.8, 45.2) n = 63	<.001 *<.001*	.74
	Learning/remembering	49.3 (48.6, 50.0) n = 623	47.5 (46.3, 48.8) n = 298	43.4 (40.9, 45.9) n = 109	<.001 *<.001*	.63
	Quality of life	49.8 (49.0, 50.5) n = 659	46.3 (45.1, 47.4) n = 313	39.4 (35.7, 43.2) n = 59	<.001 *<.001*	1.11
Healthy children	Neurosensory	48.9 (47.9, 49.8) n = 361	57.0 n = 1	31.1 n = 1	.120 *.154*	1.89
	Motor development	49.2 (48.2, 50.3) n = 333	45.6 (42.6, 48.7) n = 31	37.9 (22.6, 53.2) n = 3	.018 *.002*	1.18
	Learning/remembering	49.6 (48.5, 50.7) n = 266	46.9 (44.7, 49.1) n = 89	45.8 (39.1, 52.5) n = 14	.037 *.025*	.41
	Quality of life	50.1 (49.0, 51.2) n = 271	45.6 (43.5, 47.8) n = 86	36.2 (23.8, 48.6) n = 8	<.001 *<.001*	1.58

* first based on Anova, second based on Kruskal-Wallis non-parametric test (in italics)

### Psychosocial health by child behavior problem

Child behavior was strongly related to parental psychosocial health in both groups of parents (see Table 4). Parents whose child scored in the clinical range for internalizing and externalizing symptoms and the total problem score on the CBCL/1.5–5 had the lowest mean (i.e., poorest) MCS scores. The differences between this group and the group with children scoring in the normal range resulted in large effect sizes, indicative of clinically important differences in parental psychosocial health.

**Table 4:** Mean score, p-value and effect size for SF-36 psychosocial summary score comparing CBCL/1.5–5 normal with borderline and clinical groups

	CBCL scale	Normal	Borderline	Clinical	p-value	Effect size
NICU sample	Internalizing	49.5 (48.9,50.2) n = 841	42.5 (39.6,45.5) n = 67	40.5 (37.6,43.4) n = 78	<.001	.95
	Externalizing	48.9 (48.3,49.6) n = 925	41.6 (38.0,45.2) n = 45	35.0 (29.6,40.2) n = 32	<.001	1.43
	Total	49.3 (48.6,49.9) n = 831	43.3 (39.7,47.0) n = 34	35.3 (31.0, 39.5) n = 39	<.001	1.45
Healthy children	Internalizing	49.6 (48.6,50.6) n = 324	40.7 (36.8,44.6) n = 20	40.1 (34.0,46.2) n = 16	<.001	1.03
	Externalizing	49.3 (48.3,50.3) n = 342	43.4 (37.0,49.8) n = 14	34.1 (22.5,45.8) n = 8	<.001	1.67
	Total	49.4 (48.4,50.4) n = 330	41.5 (34.0,49.0) n = 12	36 (27.0, 44.0) n = 11	<.001	1.46

p-value based on Anova, (non-parametric tests: all p-values < .001)

### Parental psychosocial health by birth-related risk factors

Within the NICU sample, MCS score did not vary by any birth-related risk factor (i.e., gestational age; small for gestational age; apgar score; multiple birth; the presence of a major morbidity; and neonatal illness severity score), with the exception of the presence of a congenital anomaly. For this variable, MCS scores were significantly lower in parents of children with versus without a congenital anomaly (mean difference = -3.8; p = .017; effect size = -.37). Children with a congenital anomaly (n = 87) had proportionally more mild and moderate/severe health status problems in all 4 categories (see Table 5).

**Table 5:** Number (%) of NICU children with and without a congenital anomaly to report a problem for each health status category and p-value for Chi-square test of significance

Type of HS problem	Congenital anomaly	None	Mild	Moderate or Severe	p-value
Neurosensory	No	715 (95.6)	25 (3.3)	8 (1.1)	<.001
	Yes	70 (83.3)	8 (9.5)	6 (7.1)	
Motor development	No	584 (78.3)	126 (16.9)	36 (4.8)	<.001
	Yes	48 (57.1)	17 (20.2)	19 (22.6)	
Learning/remembering	No	457 (60.9)	222 (29.6)	71 (9.5)	<.001
	Yes	36 (42.9)	30 (35.7)	18 (21.4)	
Quality of life	No	481 (64.2)	233 (31.1)	35 (4.7)	<.001
	Yes	38 (44.2)	34 (39.5)	14 (16.3)	

### Correlates of psychosocial health in general

In general, variables significantly associated with the MSC score in bivariate analysis were as follows: any health status problems (mean difference = -3.8; p < .001); neurosensory problems (mean difference = -3.7; p = 0.04); motor development problems (mean difference = -4.4; p < .001); learning/remembering problems (mean difference = -2.9; p < .001); poorer quality of life (mean difference = -4.8; p < .001); more internalizing behaviour symptoms (mean difference = -8.3; p < .001); more externalizing behavior symptoms (mean difference = -9.9; p < .001); household income below $30,000 per year (mean difference = -2.6; p < .001); female gender (mean difference = -2.6; p < .001);not living as common-law or married (mean difference = -3; p = .03); more caregiver strain (r = .41; p < .001); and lower family function (r = -.44; p < .001). Borderline significance was also found for less than high school education (mean difference = -2; p = .08).

We examined a pooled model (both groups together) for a direct comparison of the NICU and healthy groups after adjustment for other variables. Due to the low number of male respondents in the healthy group, we restricted the pooled multivariable analysis to only female respondents. Predictors significantly associated with the outcome were the following: parental age (Beta = 0.15; p = 0.001); internalizing behavior (Beta = -2.06; p = 0.017); externalizing behavior (Beta = -3.24; p = 0.004); parental strain (Beta = 0.15; p < 0.001); and family function (Beta = -0.53; p < 0.001). The pooled model also showed an interaction effect between NICU admission and education (less than high school) (Beta-education = -5.94 with p = 0.009; Beta-interaction = 7.28 with p = 0.005)(see Table 6.) For the NICU group, education did not show any effect in terms of difference in outcome, but for the healthy group, lower education was associated with a significantly lower mean MCS score. More specifically, for respondents with less than high school education, the healthy group reported lower MCS scores than did the NICU group. The results were not affected by exclusion of multiple births and cases of congenital anomalies from the analysis.

**Table 6:** Beta coefficients, 95% confidence intervals, standardized beta coefficients and p-values for predictor variables in the multiple regression models for pooled model

Pooled model					
Variable	Beta	CI-low	CI-high	St. beta	p-value

Intercept	34.21	30.26	38.17		<.0001
NICU	-0.38	-1.32	0.55	-0.02	0.500
Parental age	0.15	0.08	0.23	0.08	0.001
Education	-5.94	-9.67	-2.21	-0.13	0.009
Internalizing behavior	-2.06	-3.47	-0.65	-0.07	0.017
Externalizing behavior	-3.24	-5.08	-1.40	-0.08	0.004
Caregiver strain	0.15	0.13	0.18	0.26	<.0001
Family function	-0.53	-0.60	-0.46	-0.32	<.0001
NICU-education interaction	7.28	3.01	11.56	0.14	0.005

Although other interaction terms with NICU status did not add any more significant results in the pooled model (non-significant partial F-test), we examined separate models for the NICU and the healthy baby group to further explore the association between gender and MCS score, and to evaluate the potential influence of congenital anomalies in NICU group.

### Correlates of psychosocial health for NICU sample

Variables that were significantly associated with lower MCS scores at the bivariate level include the following: female caregivers (mean difference = -3.2; p = .037); household income below $30,000 per year (mean difference = -3.3 and p < .001); not living as common-law or married (mean difference = -5.1; p < .001); neurosensory problems (mean difference = -6.44; p = .011); motor development problems (mean difference = -7.1; p < .001); learning/remembering problems (mean difference = -5.9; p < .001); poorer quality of life (mean difference = -10.4; p < .001); more internalizing behaviour symptoms (mean difference = -9.03; p < .001); more externalizing behavior symptoms (mean difference = -13.9; p < .001); the presence of a congenital anomaly (mean difference = -3.8; p = .017); more caregiver strain (r = .411; p < .001); and lower family function (r = -.441; p < .001).

Predictors that were significant in the final regression model appear in Table 7. Female gender was an independent risk factor for lower MCS score: females scored on average 5.3 points (CI interval 2.5 to 8.0) lower, which represents a moderate effect size of 0.51 (when overall NICU parents group standard deviation (SD) 10.4 for MCS was used as the denominator). Scoring outside the normal range for internalizing and externalizing child behavior symptoms independently contributed to lower MCS scores (-1.9 and -2.8, both with wide confidence intervals), with the change representing small effect sizes of 0.18 and 0.27. More caregiver strain (i.e., lower PTT) was related with poorer MCS scores. A one point change in PTT corresponded to a 0.15 (CI: 0.11–0.19) change in MCS score. In NICU parents, the mean PTT was 86.9 and SD was 18.5. Therefore, 2 SD on the PTT would represent 5.5 points on the MCS, or an effect size of 0.53. The mean score for family function (FAD) was 8.1 and the SD was 6.4. A one point change in FAD corresponded to a 0.5 (CI: 0.62; 0.42) change in MCS. Therefore a 2 SD increase in family function score (i.e., poorer family functioning) would result in a 6.4 decrease (worsening) in MCS, representing a moderate effect size of .62. Overall, the adjusted R2 was .2884 (F = 73.96; df = 5; p = < .0001), with 5 out of 15 predictors included in the full model.

**Table 7:** Beta coefficients, 95% confidence intervals, standardized beta coefficients and p-values for predictor variables in the multiple regression models for both samples

	NICU sample					Healthy baby sample				
Variable	Beta	CI-low	CI-high	St. beta	p-value	Beta	CI-low	CI-high	St. beta	p-value

Intercept	44.9	40.3	49.5		<.001	35.2	26.3	44.1		<.001
Parental age						0.3	0.1	0.5	0.1	.005
Female gender	-5.3	-2.6	-8.0	-0.11	<.001					
Education						-5.00	-0.8	-9.1	-0.1	.019
Internalizing behavior	-1.9	-0.1	-3.8	-0.06	.043	-4.0	-0.8	-7.2	-0.1	.014
Externalizing behavior	-2.8	-0.4	-5.3	-0.07	.025					
Caregiver strain	0.2	0.1	0.2	0.26	<.001	0.1	0.04	0.2	0.2	.003
family function	-0.5	-0.4	-0.6	-0.32	<.001	-0.6	-0.4	-0.8	-0.4	<.001
Quality of life						-6.9	-0.4	-13.4	-0.1	.039

### Correlates of psychosocial health for healthy baby sample

Variables that were significantly associated with poorer SF-36 MCS scores at the bivariate level include the following: younger parental age (r = .19; p < .001); household income below $30,000 per year (mean difference = -4.6; p = .005); less than high school education (mean difference = -6.22; p = 0.065); not living as common-law or married (mean difference = -6.1; p = .005); motor development problems (mean difference = -11.3; p = .043); learning/remembering problems (mean difference for any problems versus none = -2.68, p = 0.021); poorer quality of life (mean difference = -13.9; p < .032); more internalizing behavior symptoms (mean difference = -9.5; p < .001); more externalizing behavior symptoms (mean difference = -15.2; p < .018); more caregiver strain (r = .385; p < .001); and lower family function (r = -.438; p < .001).

Predictors that were significant in the final regression model appear in Table 7. The model for parents of healthy children did not include female gender (because of low numbers) and externalizing behavior symptoms, and included several variables not predictive in the NICU model (i.e., parental age; education; quality of life). Both models included internalizing child behaviors, caregiver strain and family function.

In the healthy baby sample, younger parental age was related to poorer MCS score, with a one year change in age resulting in a 0.26 (CI: 0.08; 0.45) change in MCS. A ten year difference in age would correspond to a 2.6 difference in MCS, which would represent a small effect size of 0.27 (when the overall healthy baby parent group SD for MCS (9.6) was used as a denominator). Education was also associated with MCS. Compared with high school graduates, the MCS score for parents with less than a high school education were on average 5.0 lower (CI: 0.84; 9.1), which represents a moderate effect size of 0.52, although the effect could range from minimal to large due to lower precision of the beta estimate. Child internalizing symptoms, family function and caregiver strain were associated with parental MCS in a similar way as for NICU parents. However, due to lower numbers and resulting low precision in beta estimates, the effects ranged from minimal to large. Lower parent-reported child quality of life was also associated with a lower parental MCS. Parents who reported a problem with their child's quality of life had MCS scores that were 6.9 (CI: 0.37; 13.4) lower than parents who reported at least one quality of life problem compared with those who reported at least one problem. Again, due to the small numbers, the effect could range from minimal to large. In the final regression model, the adjusted R2 was .3046 (F = 25.97; df = 6; p < .0001), with 6 out of 16 predictors included in the full model.

## Discussion

There is little information in the research literature on how parents of NICU children adapt psychologically to the demands of caregiving beyond the initial hospitalization period. We compared the psychosocial health of parents of NICU children with parents of a group of healthy full-term children using the SF-36, a popular generic measure of psychosocial HRQL. Although children admitted to a NICU at birth are at increased risk of a variety of long-term health problems, we did not find any difference in parental psychosocial health when the two groups were compared. This finding is in agreement with one of the few studies that measured mental health in parents of NICU children at preschool age. Singer et al. [17] reported that after the neonatal period, the mental health of mothers of low-risk infants did not differ from mothers of term infants, and by 3 years, they had lower levels of distress, which they suggest may be due to maternal relief after an initial period of fear and anxiety. Mothers of high-risk infants, in contrast, had more symptoms of distress at 2 years, more negative family impact at 2 and 3 years and more parental strains and illness stressors at 3 years. But by 3 years, their reported psychological distress did not differ from that of term mothers. The authors suggest that by 2 years, infant developmental scores are predictive of later outcomes, and many mothers of high-risk infants must relinquish their hopes for their children to "catch up" to healthy born children and that some psychological adaptation has taken place despite parental acknowledgment of greater family and parenting stressors. With our cross-sectional design, we are not able to confirm the trend noted by Singer, but given the lack of relationship between most birth-related risk factors and parental mental health, it is possible that the parents of high- and low-risk infants in our sample have adjusted over time.

Current health status, in bivariate analysis, was strongly related with parental psychosocial health. In both groups of parents, those whose child had a neurosensory, motor development, learning/remembering or quality of life problem had poorer psychosocial health than those with children with no problems in these areas. Child behavior was also strongly related to parental psychosocial health. More specifically, parents of children who scored in the borderline or clinical range for internalizing, externalizing and total behavior problems on the CBCL/1.5–5 reported poorer psychosocial health than parents of children who scored in the normal range. These findings were consistent across both samples of parents. The only birth-related risk factor associated with parental psychosocial health was the presence of a congenital anomaly. Here the effect size was small, but points to the possibility that a congenital anomaly may affect parents mental health adversely. Researchers have reached a consensus that a minimally important difference in HRQL is close to one half of a standard deviation [34]. The differences that we found for health status and behavior were substantially larger and therefore represent clinically important differences in parental psychosocial health. However, not all of these variables showed a significant effect in the multivariate analysis, and it is possible that these variables influence other, more proximal, variables that showed stronger effects on parental psychosocial health.

The factors associated with poorer psychosocial health in the multivariate models provide important information about correlates of adjustment for NICU and healthy baby families. In a more general pooled model, parental age, higher caregiver strain, lower family function, and child's internalizing and externalizing behavior were independently associated with poorer caregiver's mental health score. The effect of lower parental education was modified by NICU status of the child. In the healthy baby group, less than high school education indicated lower MCS score. Child externalizing behavior symptoms and female gender (parental) were associated with lower MCS scores in the NICU group, whereas lower parental age, less education and poorer child quality of life were associated with lower MCS in the healthy baby group. For both samples, as it is also seen in a pooled model results, low family function, high caregiver strain, and child's internalizing behavioral symptoms were independently associated with lower parental psychosocial health. For family function and caregiver strain, only a substantial departure from mean values (at least 2 SDs) would result in a clinically important moderate effect size for the NICU group. Our interpretation for the healthy baby sample is hampered by wide confidence intervals around the beta estimates, resulting in effect sizes that ranged from minimal to large. Internalizing behavior symptoms were associated with only a small effect on caregiver's MCS score, again with wide confidence intervals around the beta coefficients for both samples.

A recent publication outlines the integration of a number of theoretical models into one multidimensional model that can be used to describe the caregiving process [35]. This model includes the following constructs: background and context; child characteristics; caregiver strain; intrapsychic factors; coping/supportive factors; and health outcomes. Fitting our findings within this framework, we found that poorer psychosocial health in parents was associated with background/context variables (i.e., female gender, younger age, less education); child characteristics (i.e., poorer quality of life, more child behavior problems); caregiver strain; and coping/supportive factors (i.e., family function). We suggest that future research with NICU parents be conceptually based and measure constructs found in other research to be important to caregiver health.

Our study has several limitations. Because it is not possible to verify cause-effect using a cross-sectional design, we were only able to estimate the direct effect of a limited number of predictor variables on parental psychosocial health. While our study has helped to identify some possibly important caregiving variables, there are other variables important to caregiver health that we did not measure. For example, while it is possible that some parents of children with severe health problems may have received specialized or targeted services (health and/or social services) to help them cope with their child's health problems, we did not include measures to determine this. Another limitation concerns our response rate. Although it is within the range often obtained in a postal survey [36], non-response can introduce bias. Some non-respondents indicated (verbally or in writing) they were "too busy" to participate. It is also likely that some questionnaires returned to us blank were from non-English speakers. Where we had data and were able to explore response bias (NICU sample only), only a few differences in birth-related sample characteristics and outcome were found that suggests respondents had sicker babies [37]. However, our study findings about health outcomes of NICU graduates are in agreement with the larger NICU literature, so it is unlikely that the differences we found are entirely due to response bias.

## Conclusion

Our findings would suggest that overall, parental gender, family functioning and caregiver strain played influential roles in parental psychosocial health. For child characteristics, current behavior was more influential than initial birth-related risk factors.

## List of abbreviations

MCS – Mental Component Score

NICU – Neonatal intensive care unit

HS – Health status

FAD – Family Assessment Device

PTT – Parental Impact Time

## Competing interests

The author(s) declare that they have no competing interests.

## Contributions of each author

Anne Klassen contributed substantially to the study's conception and design, acquisition of data, analysis and interpretation of data; and she drafted and revised and gave final approval of the version to be published.

Shoo Lee contributed substantially to the study's conception and design, acquisition of data, analysis and interpretation of data; and revised the article critically for important intellectual content and gave final approval of the version to be published.

Parminder Raina contributed substantially to the analysis and interpretation of data; and revised the article critically for important intellectual content and gave final approval of the version to be published.

Sarka Lisonkova contributed substantially to the analysis and interpretation of data; and revised the article critically for important intellectual content and gave final approval of the version to be published.

## Pre-publication history

The pre-publication history for this paper can be accessed here:


